# Model validation: local diagnosis, correction and when to quit

**DOI:** 10.1107/S2059798317009834

**Published:** 2018-02-01

**Authors:** Jane S. Richardson, Christopher J. Williams, Bradley J. Hintze, Vincent B. Chen, Michael G. Prisant, Lizbeth L. Videau, David C. Richardson

**Affiliations:** a Department of Biochemistry, Duke University, Durham, NC 27710, USA

**Keywords:** structure validation, outlier correction, *MolProbity*, all-atom contacts, likelihood

## Abstract

Traditionally, validation was considered to be a final gatekeeping function, but refinement is smoother and results are better if model validation actively guides corrections throughout structure solution. This shifts emphasis from global to local measures: primarily geometry, conformations and sterics. A fit into the wrong local minimum conformation usually produces outliers in multiple measures. Moving to the right local minimum should be prioritized, rather than small shifts across arbitrary borderlines. Steric criteria work best with all explicit H atoms. ‘Backrub’ motions should be used for side chains and ‘P-perp’ diagnostics to correct ribose puckers. A ‘water’ may actually be an ion, a relic of misfitting or an unmodeled alternate. Beware of wishful thinking in modeling ligands. At high resolution, internally consistent alternate conformations should be modeled and geometry in poor density should not be downweighted. At low resolution, *CaBLAM* should be used to diagnose protein secondary structure and *ERRASER* to correct RNA backbone. All atoms should not be forced inside density, beware of sequence misalignment, and very rare conformations such as *cis*-non-Pro peptides should be avoided. Automation continues to improve, but the crystallographer still must look at each outlier, in the context of density, and correct most of them. For the valid few with unambiguous density and something that is holding them in place, a functional reason should be sought. The expectation is a few outliers, not zero.

## Abstract 2

An overview is provided of current crystallographic model validation of proteins and RNA, both foundations and criteria, at all resolution ranges, together with advice on how to correct specific types of problems and when you should not try so hard that you are overfitting.

## 1. Background   

Structure validation highlights the good parts and identifies possible problems in macromolecular structures (initially for X-ray crystallography, but also for neutron, NMR and cryo-EM methods). It has aspects that address (i) the experimental data, (ii) the modeled coordinates and (iii) the model-to-data fit. It was spurred into existence around 1990 after two high-profile chain mis-tracings, and started with* R*_free_ (Brünger, 1992 ▸), bond lengths and angles (Engh & Huber, 1991 ▸), twinning (Yeates, 1997 ▸), and the multi-criterion systems of *PROCHECK* (Laskowski *et al.*, 1993 ▸), *OOPS* (Jones *et al.*, 1991 ▸) and *WHATCHECK* (Hooft *et al.*, 1996 ▸). Here, we will emphasize validation of the model, from physical principles and prior experience, always within the requirement for a good model-to-data fit.

Finding problems is unrewarding unless they can be fixed, and our group has concentrated on techniques and criteria that can point the way for correction. This desire shifts the emphasis from global to local measures, and encourages cycles of validation and correction throughout the structure-solution process, not just at the end.

## 2. The foundation: all-atom contacts   

**Figure 1:**
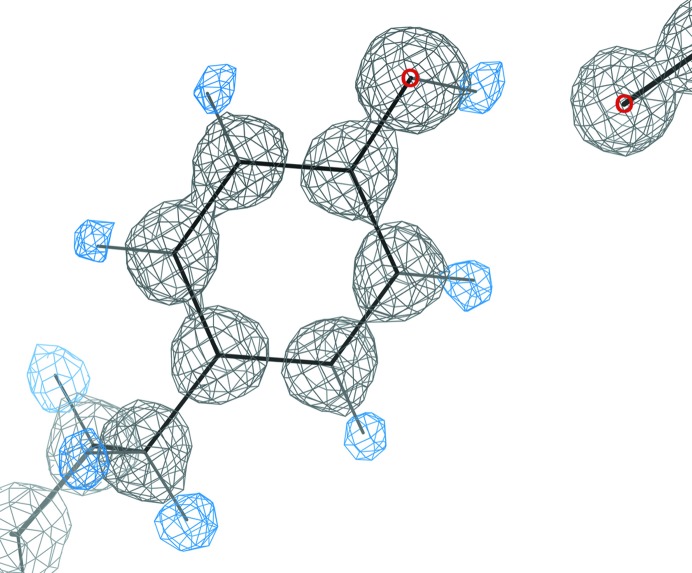
The H atoms are really there. Tyr13 in PDB entry 1yk4 for rubredoxin at 0.69 Å resolution, with 2*mF*_o_ − *DF*_c_ map (black) contoured at 1.5σ and *mF*_o_ − *DF*_c_ (blue) at 2.8σ. All H atoms are visible, including the OH donor to a nearby backbone O.

The starting point for our *MolProbity* validation is the H atom. H atoms comprise about half of the atoms in biological macromolecules, as the ‘twigs’ at the outer edges of the covalent tree structure. About three quarters of all contacts within or between molecules have an H atom on one or both sides. Historically, H atoms were seldom included because they make calculations expensive and visualizations more cluttered, and because they are not directly observable in crystallography except at extremely high resolution, since they have only a single electron, and even this electron does not diffract well. However, we know that they are really there, both from chemistry and from our very best structures (Fig. 1 ▸). All systems include some sort of ‘bump’ check, but most are simple center-to-center distance for the heavier atoms (C, N, O…) using the poor approximation of united atom radii that account for hydrogen volume but not directionality. We aim to convince the reader that explicit H atoms can be placed quite accurately from good heavier atom positions, and that considering the detailed geometry of their hydrogen bonds and steric contacts revolutionizes the ability to model and understand local structure.

**Figure 2:**
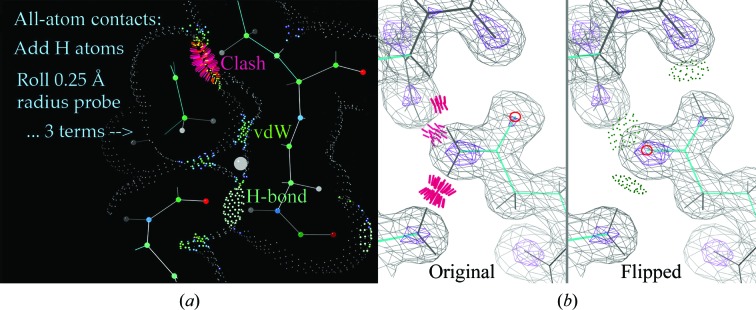
(*a*) The process of defining all-atom contacts. Small, pale dots are the van der Waals envelope of covalently bonded atoms, including H atoms. A probe sphere of 0.25 Å radius (gray) is rolled on this surface, leaving color-coded probe dots where it intersects with the surface of another atom. Favorable van der Waals contacts from just touching to 0.5 Å apart produce paired patches of green (close) and blue (more distant) contacts, while overlaps are either favorable hydrogen bonds between donor–acceptor atom pairs or repulsive overlaps in warm colors, with serious clashes of ≥0.4 Å shown by hot-pink spikes. (*b*) A definitive assignment of an amide ‘flip’ for Gln115 in PDB entry 1gk8. The larger NH_2_ group gives clashes and there are no hydrogen bonds in the original position, while the flip shows three hydrogen bonds and no clashes. The *REDUCE* flip is confirmed as correct by the higher electron density in the assigned O position.

All-atom contact analysis, as performed in *MolProbity* (Chen *et al.*, 2010 ▸), *PHENIX* (Adams *et al.*, 2010 ▸), *Coot* (Emsley *et al.*, 2010 ▸) and wwPDB validation (Berman *et al.*, 2003 ▸; Read *et al.*, 2011 ▸; Gore *et al.*, 2012 ▸), first requires *REDUCE* to be run (Word, Lovell, Richardson *et al.*, 1999 ▸). *REDUCE* adds all H atoms, at the electron-cloud center for X-ray data and at the nuclear position for NMR data (Deis *et al.*, 2013 ▸; Richardson *et al.*, 2013 ▸), and optimizes their hydrogen bonds, clashes and van der Waals contacts. It considers all possible combinations in complete hydrogen-bond networks, rotating OH, SH and NH_3_ groups but not methyl groups, and flipping Asn, Gln or His groups where needed. The first layer of waters is considered, but their H atoms are virtual and provide a donor or acceptor as needed by each interacting group. His protonation is assigned by avoiding clashes and optimizing hydrogen bonds. These strategies were chosen after extensive tests in maximizing overall accuracy by relying less on less reliably placed features, such as waters. The *PROBE* program (Word, Lovell, LaBean *et al.*, 1999 ▸) then looks at all noncovalent atom pairs whose van der Waals surfaces are ≤0.5 Å apart, calculating scores and producing dot surface visualizations, as shown in Fig. 2 ▸(*a*).

This methodology was originally tested (Arendall *et al.*, 2005 ▸), and is constantly being reaffirmed, by the near-perfect all-atom contacts seen in the well ordered parts of high-resolution structures. In less ideal circumstances the contacts act like a super-sized *R*_free_, assessing the heavier atom positions by the fit of the unseen other half: the H atoms. An extremely useful byproduct of the *REDUCE* calculation is the automated correction of Asn, Gln and His ‘flips’ and assignment of His proton­ation (Richardson & Prisant, 2012 ▸), which are easy to get wrong just from a density fit, or even if only hydrogen-bonds are considered and not clashes, since for instance an NH_2_ group is much larger than an O atom (Fig. 2 ▸ *b*). The right alternative is obvious to see and understand with this tool, but is very hard without it.

**Figure 3:**
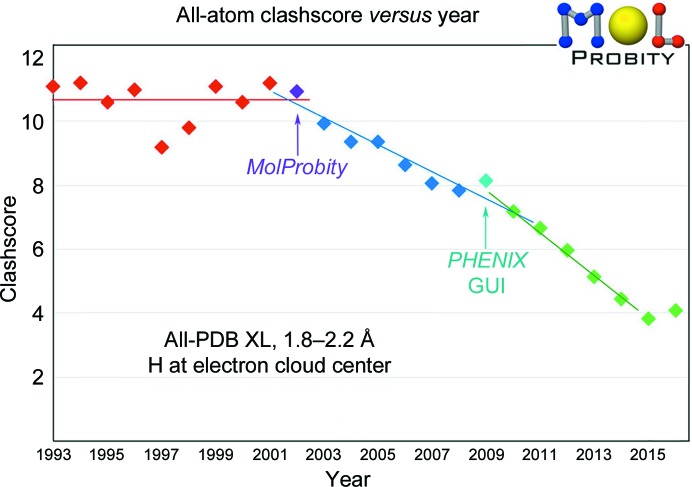
Plot of average clashscore *versus* year for mid-resolution PDB depositions worldwide, showing a steady improvement since the introduction of *MolProbity*.

Since a hydrogen bond or a clash is between a specific atom pair and is highly directional, crystallographers can see how to correct the problems that they diagnose. As plotted in Fig. 3 ▸, average all-atom clashscores have improved from 11 down to four clashes per 1000 atoms in mid-range PDB depositions worldwide since the start of *MolProbity* in 2002 (Davis *et al.*, 2004 ▸). The change has been remarkably steady, but with a few blips, including last year. It is possible that it has begun to level off: it cannot go below zero and should not actually reach zero, since our parameters are not perfect and there are usually a few small unexplained, unfixable clashes even in the best structures. The point is not to avoid all clashes by nudging something just across the 0.4 Å threshold; this does not improve biological understanding, and refinement is quite likely to nudge it right back. Instead, use clashes to find and fix places where a group was fitted into the wrong local minimum, which often does affect an interpretation. If enough of these are corrected, the map will improve elsewhere, perhaps in the active site.

## 3. Updates of traditional validation measures   

**Figure 4:**
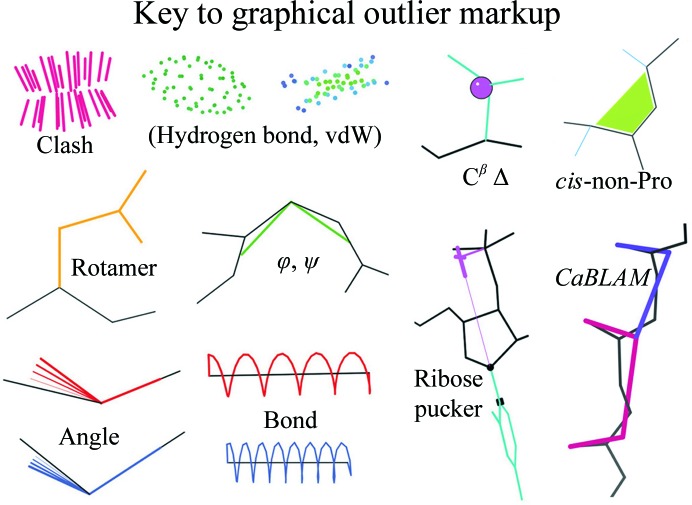
Key to the markup for various categories of validation outliers in *MolProbity*’s three-dimensional graphics, as also seen in the figures here.

Since serious problems usually show up in multiple criteria, and since it is possible to ‘game’ any one measure at the expense of the others, model validation should be as comprehensive as feasible. Fig. 4 ▸ shows a key to all of the three-dimensional graphical outlier markup in *MolProbity*, as seen on the website and in the subsequent figures. Outliers are also listed in chart or table form, with their parameters and scores. All-atom contact dots and spikes have already been explained in Fig. 2 ▸(*a*). Bond-length and angle parameters were pioneered by Engh & Huber (1991 ▸) for proteins and by Berman and Olson (Gelbin *et al.*, 1996 ▸) for nucleic acids; they are shown here as springs or fans, in red if too broad and in blue if too short. They have been updated primarily by making them context-dependent: on φ, ψ by Karplus and Dunbrack (Berkholz *et al.*, 2009 ▸), on ribose pucker by us (Jain *et al.*, 2015 ▸) or by combining angles into the C^β^ deviation, which flags a C^β^ position forced to be nontetrahedral by an incorrect local fit of either the side chain or the backbone (Lovell *et al.*, 2003 ▸). Outliers in conformation (combinations of rotatable dihedrals) are shown in gold for side-chain rotamers, in green for Ramachandran and as magenta crosses for ribose pucker. Newer validation types are designed to be more robust at low resolution (*CaBLAM*) or to flag systematic errors such as too many *cis*-non-Pro peptides. Each of the last five will be discussed further below.

**Figure 5:**
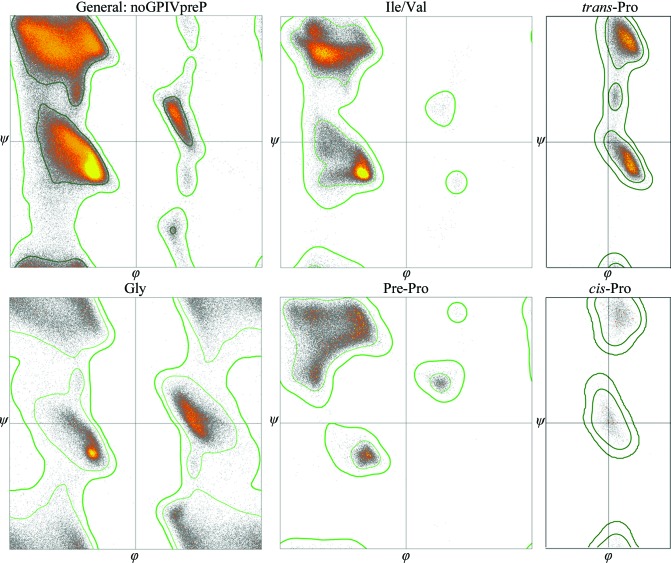
The six plots of data distributions and contours used for current Ramachandran validation. The million residues of quality-filtered data from the Top8000 data set are color-coded in 0.1° pixels, from gray for one data point to bright yellow for the highest density (30–45 data points per pixel in the general distribution). For Gly the outer contours are symmetric but the data are not, since Gly serves different functional roles in the α and the Lα regions.

The Ramachandran plot was first used for model validation by Thornton and coworkers (Morris *et al.*, 1992 ▸) and has since been updated by many groups. Current Ramachandran criteria as used in *MolProbity*, *PHENIX* and at the wwPDB (Read *et al.*, 2011 ▸; Richardson *et al.*, 2013 ▸) divide the amino-acid types into six categories, each with its own φ, ψ plot (Fig. 5 ▸). The use of reference data that have been quality-filtered both at the file level (resolution, redundancy, *MolProbity* score, ≤5% of residues with various bad outliers) and at the residue level (in this case, just no alternate conformations or backbone *B* factors > 30 Å^2^) gives plots with extremely few data points outside a smooth, reproducible outlier contour: for the general case, only one in 5000 reference examples is in this outer 60% of the plot area. These data points are mostly valid, but in poor density or at lower resolution most Ramachandran outliers are wrong. In either case, though, each outlier should be looked at. These conformations are rare because they are strained, so they are not believable without both good density and something holding them in place. The few valid outliers are very likely to be functionally important and are conserved because it is worthwhile for the molecule to spend energy stabilizing them.

Note that each of the six plots in Fig. 5 ▸ has very differently shaped contours; the other 16 amino acids show nearly identical outlier and 2% contours and so are combined in the general distribution (see the figures in the Supplementary Information for Read *et al.*, 2011 ▸). Nearly all amino acids differ significantly in their highly populated φ, ψ regions, but validation does not use this part of the distribution because we should not penalize a good loop or β-strand for not lying in the tall, bright peak of regular α-helix. For the rather different needs of prediction or design, the highly populated regions of φ, ψ or rotamer space are more important than the edges, so more narrowly divided libraries are used in that work.

**Figure 6:**
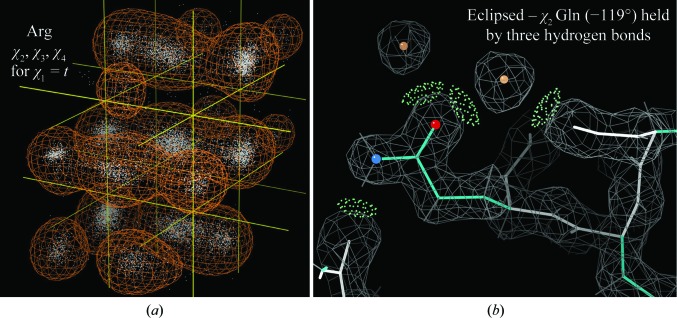
(*a*) Data-point distribution and 2% contour in the χ_2_ (into page), χ_3_ (vertical) and χ_4_ (near-horizontal) dimensions for quality-filtered Arg residues with χ_1_ *trans*. (*b*) A valid rotamer outlier: Gln321 in PDB entry 1n83, with its eclipsed χ_2_ (−119°) held by three side-chain hydrogen bonds and clearly validated by the electron density.

Side-chain rotamers are another traditional (Ponder & Richards, 1987 ▸) but recently updated protein validation criterion. Side chains have between one and four χ dihedral angles, and their rotamer distributions must be treated as multidimensional because the occurrence probabilities are not just a product of the individual χ preferences. Fully tetrahedral χ values are quite cleanly trimodal, while χ values between a tetrahedral and a planar group are clustered but more continuous and often unevenly spaced, as seen for Arg χ_4_ in Fig. 6 ▸(*a*). Even for the all-tetrahedral case the barriers to rotation are only about 3 kcal mol^−1^, so two or three hydrogen bonds can hold an eclipsed χ angle in place, as for the Gln in Fig. 6 ▸(*b*), or tight packing can hold an eclipsed χ angle for an aromatic ring. However, a side chain out on the surface without strong inter­actions should be fitted as a good rotamer, or better yet as two or three alternates in good rotamers.

Our most recent rotamer update (Hintze *et al.*, 2016 ▸) is in *MolProbity* and *PHENIX* but not yet in wwPDB validation. It took advantage not only of more high-resolution structures but also of the PDB requirement since 2008 for data deposition, in order to improve the residue-level quality filters. A combination of 2*mF*_o_ − *DF*_c_ electron-density values, real-space correlation and *B*-factor measures produced a large reference data set with very few false positives (that is, data points that were unconvincing on manual inspection). This allowed us to define well behaved outlier contours that exclude only 0.3% rather than the previous 1% of the reference data, and to add a 2% contour defining allowed *versus* favored rotamers, analogous to standard practice for Ramachandran measures.

## 4. Typical corrections   

**Figure 7:**
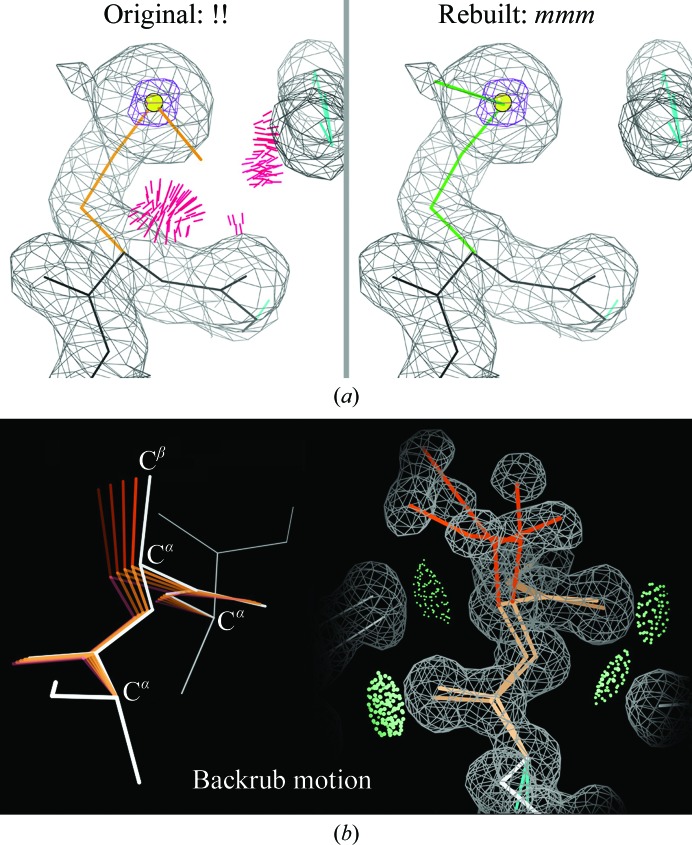
(*a*) An easy outlier correction. The methyl of Mse351 in PDB entry 1j58, as deposited, shows several bad clashes and the side chain is a rotamer outlier. The favorable *mmm* rotamer fixes both problems. (*b*) The backrub motion, shown as a schematic of the small-amplitude backrub rotation around the C^α^ *i* − 1 to *i* + 1 axis with leverage on the C^α^—C^β^ direction and on side-chain contacts, and an example of alternate conformations in Ile47 of the 1n9b β-sheet, where the backrub shift allows good packing in two distinct rotamers.

A misfitting inside a molecule usually produces outliers in more than one criterion. A bad clash means that one or both of the clashing atoms is in the wrong position, but it is seldom the case that just moving the two atoms apart is the right answer. For instance, for the selenomethionine (Mse351) in Fig. 7 ▸(*a*) all of the clashes have the methyl group in common, and the side chain is a rotamer outlier. Among rotamers that keep the Se atom centered in its clear density, the *mmm* rotamer (−60, −60, −60°) fits with no clashes and even places the methyl in a tiny bit of electron density, providing a win–win answer.

For rotamer problems that shift more of the side chain, the subtle but powerful ‘backrub’ motion (Davis *et al.*, 2006 ▸) is usually needed. It is approximated by a rigid rotation around an axis between the *i* ± 1 C^α^ atoms, with small reverse rotations of the two individual peptides if needed to maintain their hydrogen bonds or to keep the carbonyl O atoms in density (Fig. 7 ▸ *b*). A backrub is the most common movement used by protein backbone to accommodate alternate side-chain conformations that change the rotamer or hydrogen bonding (or even mutations; Keedy *et al.*, 2012 ▸), using the leverage of a very small backbone shift in the C^α^—C^β^ bond-vector direction to make substantial changes in side-chain contacts. This same motion is amazingly effective in correcting a misfitted side chain. The backrub correction can be directly controlled in the *KiNG* graphics and modeling program (Chen *et al.*, 2009 ▸; Richardson & Headd, 2012 ▸), providing a better understanding of how it works within the automated side-chain rebuilding in *Coot* or *PHENIX*.

**Figure 8:**
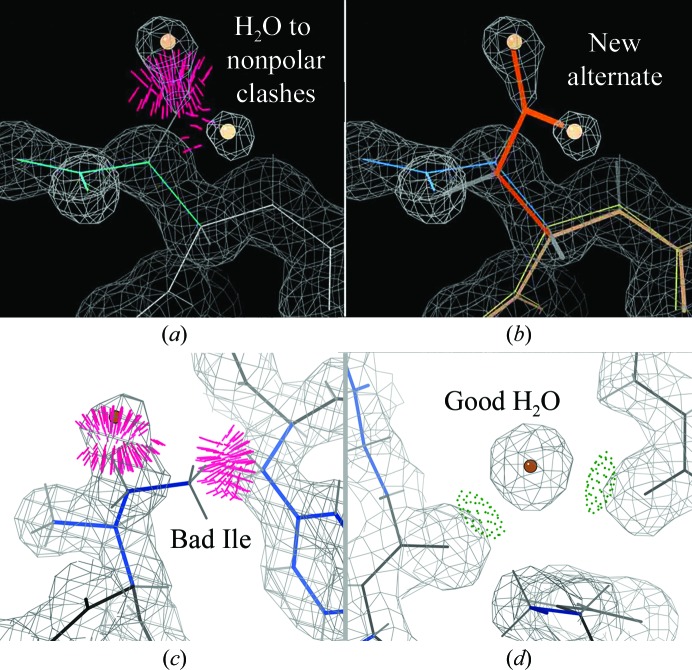
Interpreting ‘water’ peaks. (*a*) When they clash with nonpolar atoms then they are likely to be the next atoms in an unmodeled alternate (*b*), as shown for Asp9 in PDB entry 1eb6. (*c*) Do not let a ‘water’ push a side-chain atom out of its density, as happened for Ile195 in PDB entry 3js8. (*d*) A good water peak should show density separated from other atoms, with at least one polar interaction at good hydrogen-bond distance; here, HOH 543 in PDB entry 3js8 makes two good hydrogen bonds. Contours are at 1.2σ.

Another very common type of correction that is needed is finding the right model interpretation for a problematic density peak fitted as a water (Headd & Richardson, 2013 ▸). If the ‘water’ is really an ion it will show all-atom clashes rather than hydrogen bonds to polar groups, and the charge of these groups will indicate the complementary charge of the ion. If several ‘waters’ are too close together within continuous, interestingly shaped density, they probably represent an unidentified small-molecule ligand. If it proves impossible to figure out what the ligand is, it would be a great help to future users of the structure to give the group the UNL (UNidentified Ligand) designation provided by the PDB. If a ‘water’ with low or no density clashes with an outlier residue, it may be left over from a difference peak next to a misfitting such as a backward-fitted C^β^-branched side chain, and correcting the side chain and deleting the water may give an excellent answer. If the ‘water’ has clashes with non-outlier nonpolar atoms, as in Fig. 8 ▸(*a*), it is almost always the next atom in an unmodeled alternate conformation; trying additional rotamers, with backrub as needed, may identify a perfect fix such as the Asp in Fig. 8 ▸(*b*). As also noted below, a water clash with an alternate conformation means that the water needs a lower occupancy, and preferably an alternate flag as well. The case, one hopes, that will not occur in your more careful structure is where a ‘water’ was fitted early on into the density of a side-chain atom and forced this side-chain branch out of place. As in Fig. 8 ▸(*c*), this type is immediately clear once anyone actually looks at it. Finally, the peak may of course really be a water molecule, in which case it should have a peak well separated from the macromolecule, with at least one polar interaction at good hydrogen-bonding distance, as for the water in Fig. 8 ▸(*d*).

## 5. High resolution   

**Figure 9:**
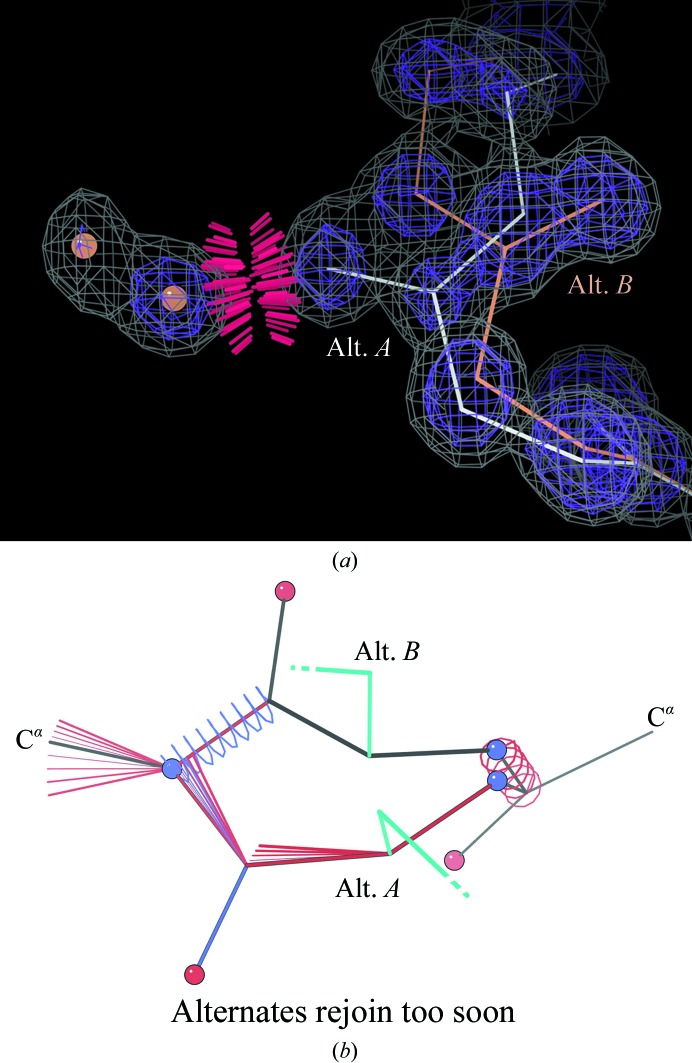
Handling alternate conformations. (*a*) Use peak heights to assign consistent alternate IDs, including partial occupancies for interacting waters, which was not performed here. Leu105 from PDB entry 1gwe at 1.2 and 3σ. (*b*) When any alternate backbone atom is widely separated, do not rejoin alternates until the flanking C^α^ atoms in order to avoid bad geometry. Here, for Asp42 from PDB entry 1w0n, there are bond-length outliers up to 8σ and bond-angle outliers up to 12σ.

High resolution is wonderful, but complex, and requires much more work than one might expect. The hardest part is correctly sorting out the many alternate conformations that are visible. Even when each atom position shows a distinct peak, they often cross back and forth confusingly, making it hard to trace more than one valid, self-consistent model through them. Historically, the problems are worst in alternates *B* and higher, since most validation systems have not evaluated these at all. *MolProbity* has always given some assessment of multiple alternates, and we are now in the process of making this functionality more complete and easy to use. Some advice in the meantime is that if the occupancies are discernably different, use the relative peak heights to join up atoms. Clashes with neighboring groups often mean that they also need modeled alternates, perhaps not differing enough to have been obvious. Nearby waters are the most frequent offenders, as in Fig. 9 ▸(*a*), since they are really part of the alternate conformation network but are typically only given high *B* factors rather the partial occupancy and alternate flags that they really deserve. Watch out for deviant bond-length, angle and C^β^ deviations, which signal that the alternates are either incorrectly mixed or that separate alternates were not defined widely enough along the covalent structure. If alternates for a side chain are fitted with C^β^ atoms >0.2 Å apart then alternates should also be defined in the backbone, and if any alternate backbone atoms are widely separated then the alternates should not rejoin at the peptide bonds (as is typically performed), but only at the flanking C^α^ atoms. *PHENIX* can now extend alternates in this manner (Deis *et al.*, 2013 ▸) far enough out to avoid the sort of dire geometry seen in Fig. 9 ▸(*b*). In another program, try duplicating and alternate flagging the few extra atoms that need to separate slightly.

A problem that is easier to avoid is globally downweighting the geometry term at high resolution, so that loops or termini in poor density can end up with near random-like coordinates, >1 Å clashes and >20σ bond-length and angle deviations (Chen *et al.*, 2011 ▸). Always look at your very worst outliers before deposition!

## 6. Tackling low resolution   

**Figure 10:**
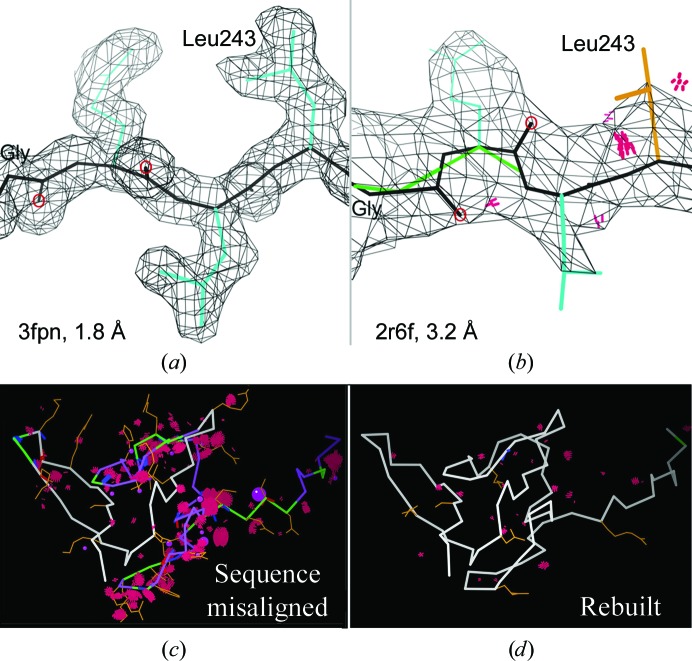
Difficulties at low resolution. (*a*) Higher resolution shows that this is a regular β-strand with no outliers or clashes, but in (*b*) the backbone CO directions are misoriented because they are not observed, and the side chains are pulled inwards towards density nubbins. Contours are at 1.2σ. (*c*) Localized outliers for a long sequence misalignment in 70S ribosomal protein L27 at 3.2 Å resolution (PDB entry 3i1n). (*d*) After rebuilding of the one- to three-residue sequence shifts in the improved PDB entry 4gd1 (Dunkle *et al.*, 2011 ▸).

There is no question that low resolution (≥3 Å) is a truly difficult challenge. Discernable bumps for carbonyl O atoms mostly disappear, giving ill-defined backbone conformations. Some side-chain atoms should genuinely lie outside the density, not only confusing rotamer choice but also tempting both people and software to scrunch them back in. Electron-density connectivity is part way along in its change from following the atomic connectivity at 2 Å to being a slab for β-sheet and a solid tube for α-helix by 5–6 Å, and it makes this change through inconsistent, misleading intermediate forms. Crystallographic methods were developed at resolutions where one can first trace the chain and then deal with side chains, but at low resolution they mix together, with the size and position of local side chains causing backbone density to break or coalesce in the wrong patterns. The best overall advice is always to fit structure as much more regular and ideal than it looks, which essentially always turns out to be the right answer if a higher resolution structure is solved later. For instance, Fig. 10 ▸(*a*) shows that the blobby bit of structure in Fig. 10 ▸(*b*) is actually a very regular β-strand with full hydrogen bonding, good rotamers and no Ramachandran or clash outliers. Once regular stretches of secondary structure have been fitted, they can be bent or shifted somewhat better into the overall density by refinement tools such as *DEN* (Schröder *et al.*, 2010 ▸), jelly body (Murshudov *et al.*, 2011 ▸) or morphing (Terwilliger *et al.*, 2012 ▸), but preferably with the help of judicious hydrogen-bond restraints to minimize distortion.

Beware of sequence misalignments, which hurt biological interpretation by misplacing functional side chains large distances from where they should be, and which are very much more common at low resolution than anyone would like to believe. Figs. 10 ▸(*c*) and 10 ▸(*d*) show a before-and-after portrait for the longest of about 20 misalignments that we have worked on (Dunkle *et al.*, 2011 ▸). When you find the right offset, the improvement is sometimes magical.

**Figure 11:**
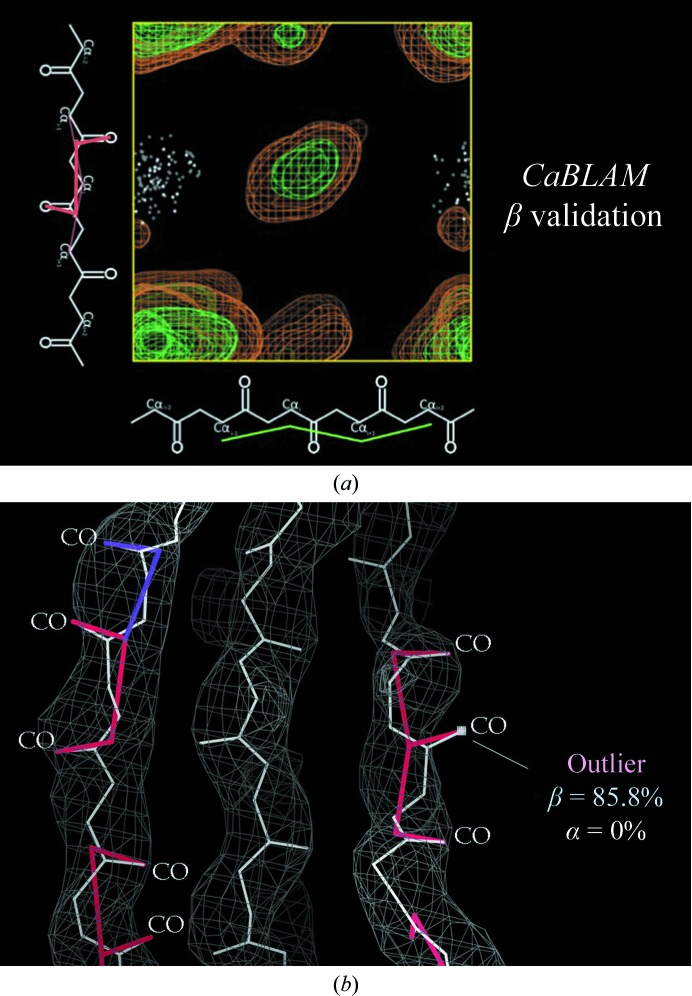
*CaBLAM* low-resolution diagnosis. (*a*) Plot of CO *versus* C^α^-in virtual dihedrals, with contours for the good β-sheet reference data and white data points for examples that have three adjacent CO bonds parallel rather than alternating. (*b*) *CaBLAM* scoring for three such outliers, showing definitively that they should be fitted as regular β structure. PDB entry 3i1n; contours at 1.2σ.

To more easily and reliably locate helices and strands at low resolution, there is a new tool called *CaBLAM* (*C^α^-Based Low-resolution Annotation Method*; Williams *et al.*, 2013 ▸). It defines a novel parameter space of C^α^–C^α^ and CO–CO virtual dihedrals, where the CO dimension diagnoses fitting problems caused by the frequent large distortions of peptide orientation at low resolution and the two C^α^ dimensions identify the probable secondary structure disguised by these problems. The C^α^ trace is the most reliable feature in low-resolution models, whereas peptide orientations are often very incorrect, destroying the use of φ, ψ Ramachandran values or backbone hydrogen bonds for identifying secondary structure. Fig. 11 ▸(*a*) shows a plot of the CO *versus* C^α^-in virtual dihedrals with contours for β structure in the good reference data. The white data points, which are well outside the 99% contour, represent >100 cases of a systematic error that is fairly common at low resolution, where three CO bonds in a row point in the same direction rather than alternating. Fig. 11 ▸(*b*) shows a ribosomal protein example of this, where *CaBLAM* diagnoses outliers and an 85% likelihood of disguised β structure, guiding corrections that lowered all types of outliers and improved the hydrogen bonds and *R* factors. *CaBLAM* validation is reported in *MolProbity*, and is available in *PHENIX* as one of the diagnostics for secondary structure.

## 7. RNA   

RNA structure has quite different properties than either DNA or protein, and its complex tertiary structures, catalytic and binding functions, and roles in large dynamic molecular machines make it highly important. RNA backbone conformation is crucial to all of these functions, but has too many variables to model straightforwardly at the usual resolutions attainable, where the backbone between ribose and phosphate is a featureless tube. Fortunately, there are some tools to help with this problem (Jain *et al.*, 2015 ▸).

**Figure 12:**
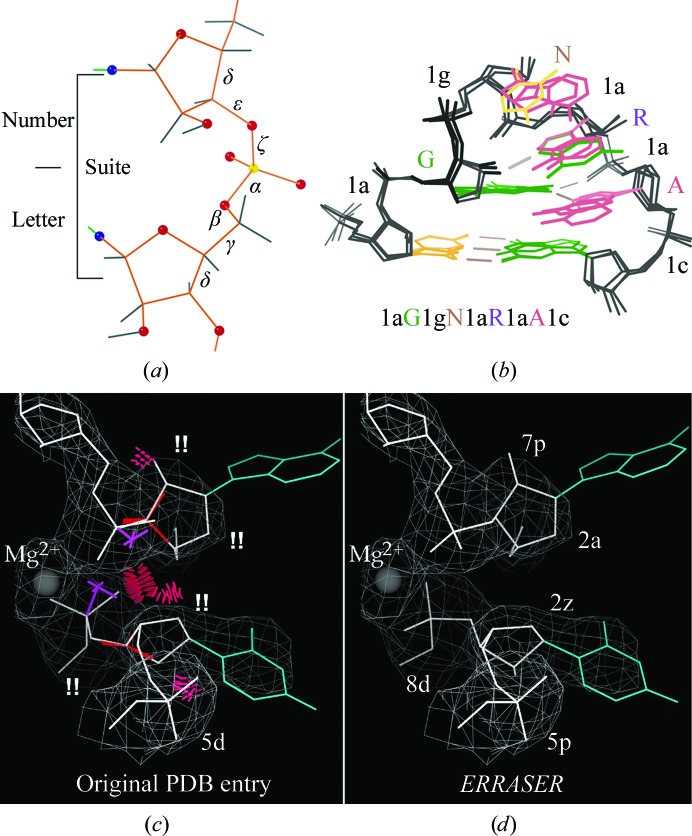
RNA backbone conformers and corrections. (*a*) Definition of the suite divisions (sugar-to-sugar) of RNA backbone. (*b*) Using two-character suite names to describe the backbone conformation of the GNRA tetraloop. (*c*) Original conformation of two touching loops in the riboswitch (PDB entry 2gis) at 2.9 Å resolution, with clashes, bad ribose puckers and four out of five outlier suite conformers(!!). (*d*) After correction using *ERRASER*.

Dividing RNA backbone into sugar-to-sugar units (suites) works better than using chemical nucleotides and shows that the RNA backbone is ‘rotameric’ (Murray *et al.*, 2003 ▸), while that of DNA is not. As an international collaborative effort (Richardson *et al.*, 2008 ▸), we defined a set of about 50 distinct, valid RNA backbone conformers across the seven variable dihedrals in a suite, each with a two-character name (Fig. 12 ▸ *a*). The GNRA tetraloop conformation is described in this system as 1a**G**1g**N**1a**R**1a**A**1c (Fig. 12 ▸ *b*), and the conformers can be used in model building. Even simpler and more powerful is the ‘P-perp’ test for 2′-*endo* *versus* 3′-*endo* ribose puckers. At most resolutions the ribose pucker cannot be seen. Fortunately, however, it turns out to be determined by two features that can be seen in the density: the phosphates and the direction between the ribose and base, which is the line of the glycosidic bond. The P-perp test drops a perpendicular to this line from the 3′ P; if the perpendicular is long (≥3 Å) the ribose pucker is 3′ and if it is short the pucker is 2′. This difference is easy to see by eye when manually fitting, and it is performed on the fly in *PHENIX* refinement to allow use of pucker-specific target parameters.

Correcting the whole backbone conformation is a very hard search problem. Rhiju Das, with our encouragement, developed an automated protocol called *ERRASER* (Chou *et al.*, 2012 ▸; Adams *et al.*, 2013 ▸). It diagnoses clashes and incorrect puckers with *MolProbity*, uses *relax* in *Rosetta* and a special-purpose brute-force search within *Rosetta*, followed by *Rosetta* and *PHENIX* refinement, and repeats this cycle. It requires the installation of *Rosetta* and takes a large amount of computer time, but produces excellent results and truly beats weeks of rebuilding and usually failing. As an example, Figs. 12 ▸(*c*) and 12 ▸(*d*) show a noncovalent contact of loops in a riboswitch with an Mg atom (currently *ERRASER* does not see non-RNA atoms); it fixes all clashes and pucker outliers, all suite conformer outliers and even improves the Mg coordination. Its corrections are not always quite this thorough, but it is amazingly effective. If one is having serious problems with an RNA structure, *ERRASER* should really be tried.

## 8. Avoiding systematic errors by model building with likelihood   

**Figure 13:**
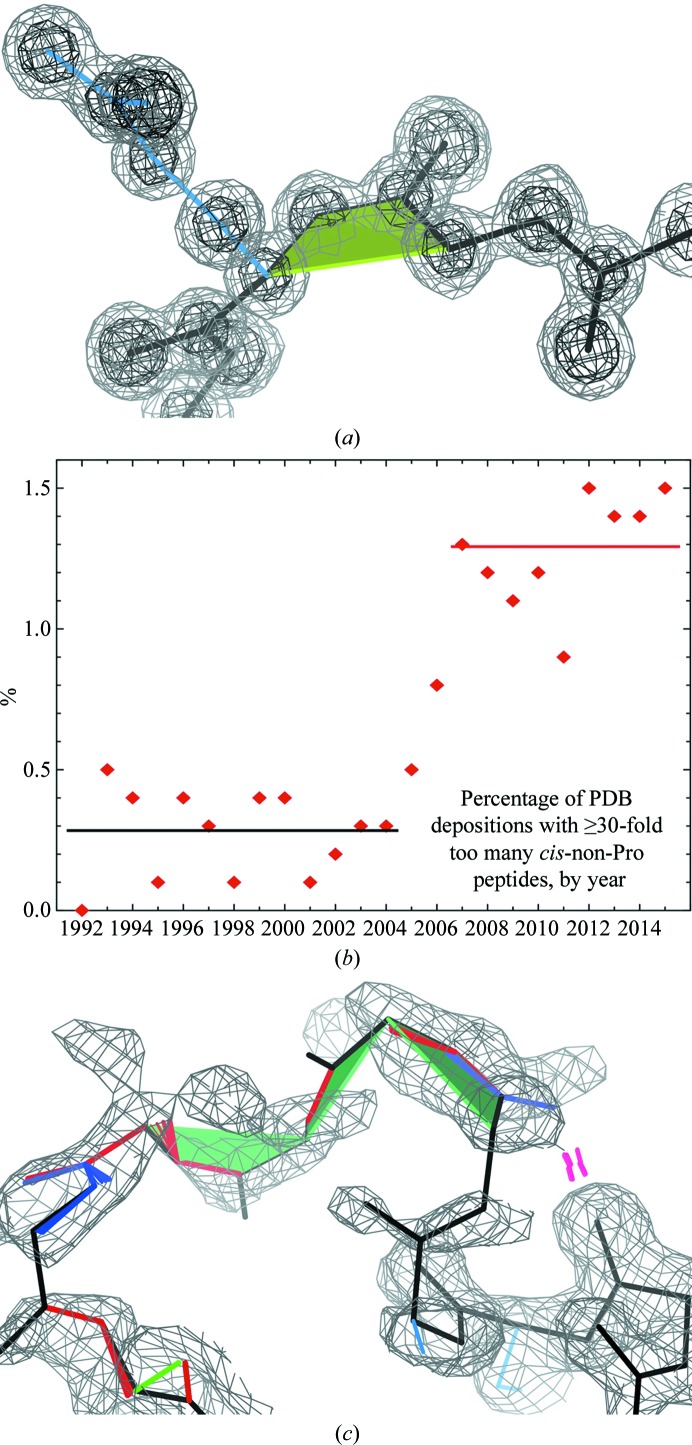
Use and overuse of very rare *cis*-non-Pro peptides. (*a*) A clear, genuine *cis*-non-Pro in PDB entry 2ddx at 0.86 Å resolution, flagged by the seagreen trapezoid. (*b*) Time-course plot of the epidemic overuse of *cis*-non-Pro peptides. (*c*) An example of how *cis* peptides can fit better than *trans* peptides into patchy, poor electron density at 1.2σ. PDB entry 2j82, 1092 loop.

Most of this validation has been around for quite a long time, and one might think that all one needs to do is check out the sliders and worst specifics on the PDB report. However, new issues keep arising from new methodologies or from unusual things in new macromolecules. Much of validation is now built into automated procedures, which is great, but occasionally this makes something new go wrong. The most notable current example is with *cis*-non-Pro peptides. As seen in Fig. 13 ▸(*a*) there are indeed genuine examples, which are almost always functionally important, but they are very rare: only about one in 3000 nonproline peptides are *cis*. However, as first pointed out by Croll (2015 ▸), in the last ten years there has been an epidemic of their overuse, with >10% of structures containing ≥30 times too many *cis*-non-Pro peptides (Fig. 13 ▸ *b*), probably without the depositors realising. Once a conformation is in lists or fragment libraries, it becomes used whenever it happens to fit just a little better, and overall usage is not tracked. The *cis*-non-Pro case is even worse than random, since a *cis* peptide is more compact than a *trans* peptide and fits better into patchy density or contracted density in a low-resolution loop (Fig. 13 ▸ *c*). The first, easy stopgap is that *cis*-non-Pro peptides are now prominently flagged (see Fig. 4 ▸; Williams & Richardson, 2015 ▸) in *MolProbity*, *PHENIX* and *Coot* so that people will be aware of them. Highly twisted peptides (ω > 30° nonplanar) are also now flagged; they are essentially never correct, since only two clear examples of >30° have been found in good reference data (Berkholz *et al.*, 2012 ▸).

However, we really want to prevent these in the first place, rather than needing to correct them later. Such overuse occurs because conformations or fragments are chosen one at a time, only considering local density fit, with no weighting by probability of occurrence. For low-resolution crystallography or poor electron density, we need a Bayesian likelihood approach to model building. This means balancing the relative prior probability (frequency of occurrence) against the relative likelihood, for each alternative, that it could produce the observed density or data.

Our test implementation of this strategy (i) defines prior probability as the log of occurrence frequency in our laboratory’s reference data set, currently the Top8000 data set, filtered by clashes, electron density and other quality criteria (Hintze *et al.*, 2016 ▸) and (ii) defines data likelihood by the reciprocal-space likelihood function (Read, 1986 ▸, 2001 ▸) that has been successful in refinement and molecular replacement. Different proposed conformations (for example *cis versus trans*) are compared by running carefully equivalent parallel refinements and balancing the resulting log-likelihood scores with the prior probabilities.

**Figure 14:**
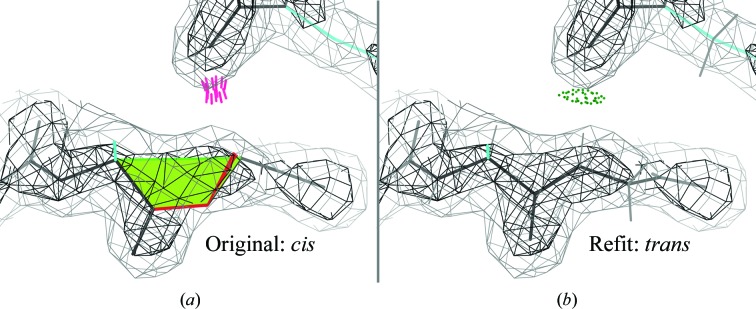
Likelihood-based choice of *cis versus trans* peptides. (*a*) Original *cis* model of Lys–Gly270 in PDB entry 2cn3, with a decent fit to the contours at 1.2 and 3σ. (*b*) Model rebuilt as *trans*, with even better fit, a hydrogen bond rather than a clash, and a log-likelihood gain of 75.6, plus eight units better log prior probability.

The results so far are surprisingly sensitive. Cases where the correct answer is truly obvious give log-likelihood differences in the hundreds, so no priors are needed. The more subtle example of the Lys–Gly *cis*-peptide in Fig. 14 ▸(*a*), which is a decently acceptable fit in its density, gives a worse LLG than the *trans* rebuild (Fig. 14 ▸ *b*) by 75.6. Adding the prior-probability disadvantage of 8 (log of 3000:1) prefers the *trans* form by 83.6, while *R* and *R*_free_ are unhelpful (±0.0001). This unambiguous answer is confirmed by the correction of a bond-angle outlier and the change of a clash to a hydrogen bond, neither of which are seen by the Bayesian calculation. In even more demanding tests at 3.5–4 Å resolution, the ΔLLG was in the range of −2 to +10 in favor of what we believe to be the correct answer, allowing the prior probability to make the choice clear. In contrast, real-space fit measures can show strong but incorrect preferences (such as for squeezing all atoms into density). After this proof-of-concept, we are now working on implementing this method in practical, production-level model building, so we hope that this type of validation outlier will become avoidable with less effort.

## 9. Conclusions: when to stop   

Here are the main take-home points for your own work.

**Figure 15:**
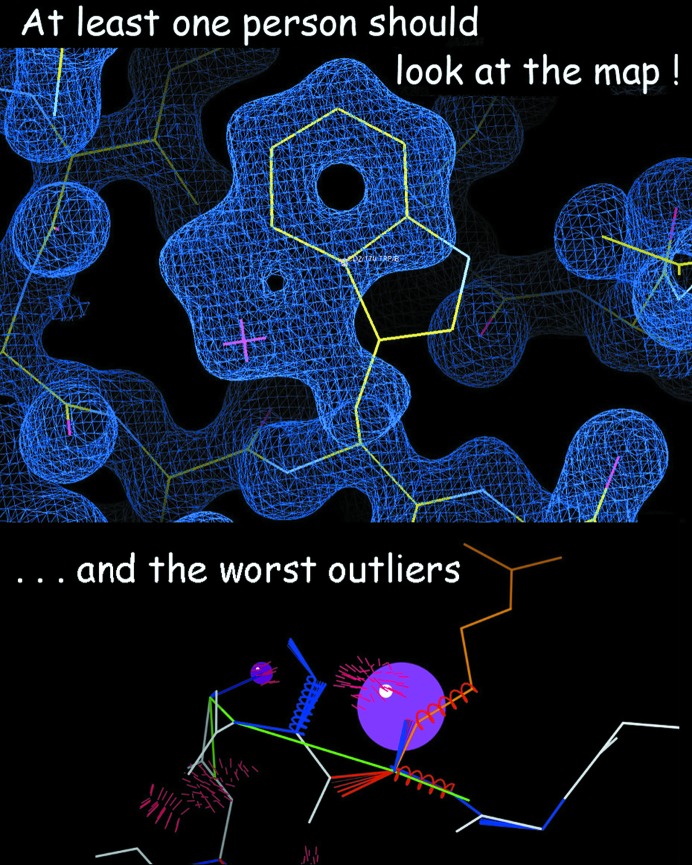
A take-home message.

(i) At least one person should look at the map and at each of the worst outliers (Fig. 15 ▸).
(ii) The goal is few outliers, not zero.
(iii) Follow the Zen of model anomalies.
  (1) Correct most of them.
  (2) Treasure the genuine few.
  (3) Then rest serenely.
